# An Overview of Biorefinery Waste for Microbial Production of Green Plastic in a Circular Economy^§^

**DOI:** 10.17113/ftb.63.02.25.8966

**Published:** 2025-06

**Authors:** Geethika Gudapati, Sridevi Veluru, Tanmayi Bora, M Tukaram Bai, Anupama Kavya Priya Dwarapureddy, Giri Prasad Reddi, Husam Talib Hamzah

**Affiliations:** Department of Chemical Engineering, Junction, AU North Campus, Andhra University North Campus, Andhra University, Visakhapatnam, 530003 Andhra Pradesh, India

**Keywords:** biorefinery, fermentation, bioplastics, circular bioeconomy

## Abstract

An increasing amount of plastics is being used due to the growing population. Plastic waste pollution has become a major problem, especially in the marine environment, due to the increasing global demand for plastic materials. Bioplastics produced from waste in biorefineries offer a sustainable alternative to traditional plastics by recycling materials that are normally thrown away in the food, farming and manufacturing industries. This technology tackles both the plastic waste crisis and the inefficient use of biomass. By recycling biorefinery waste into bioplastics, the impact on the environment can be reduced, waste minimised and less fossil fuel consumed. Improving material qualities, reducing production costs and optimising the efficiency and scalability of these processes are all ongoing challenges. This review focuses on waste biorefineries for bioplastic synthesis as a sustainable approach to the circular bioeconomy. It also provides a better understanding of environmental sustainability, societal well-being and technological advances in the utilisation of various biorefineries as different substrates and methods for bioplastic synthesis.

## INTRODUCTION

As the world's population is growing, the use of plastics in trade, industry and households remains significant. Statistics predict a population of 9.7 billion by 2050 (*1*). Growing disposable incomes, technological possibilities and population size will increase the demand for polymer products. The production of plastics is expected to exceed 1.1·10^9^ kg (*2*) by 2050 and 155–265 metric tonnes per year of untreated plastic waste will be generated worldwide by 2060 (*3*). Plastics are popular because they are inexpensive, lightweight, resistant to microorganisms and thermally and chemically insulating (*4*). The production and incineration of these polymers emits dioxins, carbon dioxide and methane into the air, contributing to the depletion of fossil fuels. An increase in greenhouse gas emissions has an effect on the environment (*5*). The non-biodegradable nature of plastics and negligent disposal on land and in the sea have led to a continuous increase in waste pollution and its harm to humans and aquatic life. The accumulation of plastic waste is a major, unsolved environmental problem.

A possible strategy to combat plastic waste is the production of biopolymers from biorefinery waste. The production of ’biodegradable plastic’ or ’bioplastics’ is a feasible alternative strategy that can be developed to solve this problem (*6*). Increasing amounts of biodegradable polymers from renewable sources are gradually replacing their synthetic counterparts (*7*-*9*). Bioplastics are produced from renewable biological sources rather than fossil fuels to prevent depletion of scarce resources. These sources include plants, biowaste or microbes (*10*). The harmless nature of bioplastics, their biocompatibility and rapid decomposition without harming the environment have led to a sharp increase in demand for them worldwide (*11*).

The total amount of bioplastic produced in 2021 was roughly 2.36 million tonnes, of which 1.55 million tonnes were biodegradable and 0.86 million tonnes non-biodegradable (*12*). While most reviews focus on other assessments, this review explores the alternative bioplastics generated from waste materials such as lignocellulose, algae, sugarcane waste, municipal sewage and food sector leftovers, rather than traditional feedstocks like corn starch and sugarcane. We will also discuss how artificial intelligence (AI), the internet of things (IoT) and machine learning (ML) can be used to improve the process of producing bioplastics from algae. Analysis of the improvements in biodegradability, sustainability and conversion technology shows that waste valorization and smart technologies are the main drivers for the industrial production of bioplastics. Compared to typical synthetic plastics derived from petroleum, the production costs of bioplastics made from renewable sources are one to one and a half times lower (*13*). The United Nations has proposed a set of sustainable development goals that bioplastics could help to achieve in the future. These goals include using less hazardous chemicals in manufacturing, discovering innovative methods for recycling and degrading materials, and minimising reliance on fossil fuels, particularly through the use of circular economies (*14*, *15*).

Climate change, pollution, global warming, depletion of fossil resources and waste disposal are some of the long-standing environmental problems that have significantly worsened and are usually the consequence of harmful human activities. A low-carbon economy, in which the circular bioeconomy plays a vital role, is important to find an immediate solution to the world's main challenges. The production of biopolymers and other strategies used by waste biorefineries are one possible action.

The term ’circular bioeconomy’ is a combination of the principles of the circular economy and the bioeconomy and characterises this hybrid approach. It implies that biomass from biological resources is systematically utilised to boost the economy. The efficient use of biomass, including waste and side streams, to sustainably produce high-value items (such as food, biomaterials, feed and bioenergy) is the promise of a circular bioeconomy. To develop useful bioproducts, this method focuses on recycling, reusing and remanufacturing while maintaining a sustainable production process. The circular bioeconomy could be considered an example of a low-carbon economy because it promises a more environmentally friendly and long-term sustainable future (*16*-*19*).

Biorefining is one of the most important solutions for promoting the bio-based circular economy, which closes the cycle of fresh or raw resources, water, minerals and carbon. By outlining the drawbacks of bioplastics and discussing the potential contribution of biodegradable plastics to the fight against global plastic pollution, this review provides a comprehensive examination of whether bioplastics solve the problem or are just an example of ’greenwashing’ to make products look good for the environment. Unlike conventional studies that focus on primary feedstocks such as corn starch and sugarcane, this review emphasises alternative waste-based sources, such as lignocellulose, algae, sugarcane waste, municipal sewage and food industry residues. Furthermore, it integrates emerging technologies such as AI, IoT and ML to optimise bioplastic production, an underexplored area. Another purpose of this review is to discuss how microbial bioplastics produced from different types of waste could contribute to a circular bioeconomy.

## TYPES OF BIOPLASTICS

Polymers are long chains of monomers or repeating units linked to make polymers. Polymerization, polycondensation and polyaddition processes involving fossil fuels are the typical ways of manufacturing these macromolecules. There is a growing interest in competitive biodegradable materials to minimise waste and pollution. Bioplastics are a new form of plastic produced by microorganisms or renewable feedstocks. They come from a biological system and represent a more sustainable future by significantly reducing energy usage and the greenhouse effect.

A wide range of biological resources, including plants, algae, fungi, bacteria and organic waste, are utilised to manufacture bio-based plastics. Polymers produced by microorganisms and plants are the raw material for the production of bio-based polymers (*20*, *21*). Since the 19th century, cellulosic materials—the most common organic compounds and primary component of plant tissue—have been used (*22*). Bioplastics can also be produced from natural sources using synthetic processes (*23*, *24*). Typically, three basic methods are used for the production of bio-based plastics: *i*) bio-monomer polymerization, *ii*) modification of polymers that already exist, and *iii*) extraction from microorganisms. The bioplastic can be produced from biomass products, genetically modified microorganisms using biotechnological approaches and petrochemical compounds.

Polylactic acid (PLA), polybutylene succinate (PBS), cellulose acetate (CA), starch-based polymers (SBP), bio-based polyethylene terephthalate (bio-PET) and bio-polyethylene (bio-PE) are the most well-known bio-based plastics. The important properties of these six bio-based plastic types are described in Table 1 (*25*-*29*).

**Table 1:** Types of bio-based biodegradable plastics

Bio-based plastic	Conversion	Application	Reference
Polylactic acid (PLA)	PLA is produced by fermentation of plant carbohydrates by different bacterial species	• Agriculture • Tissue engineering • Biomedicine	(*25*)
Polybutylene succinate (PBS)	PBS is produced by hydrolysis of non-edible lignocellulosic biomass	• Biomedicine • Hygiene products • Biodegradable bags • Mulch film	(*26*)
Cellulose acetate (CA)	Cellulose reacts with acetic acid and acetic anhydride in the presence of a catalyst, resulting in cellulose acetate	• Textile industries • Plastic films	(*26*)
Starch-based polymers (SBPs)	SBPs are substances made from native starch in its granular form	• Packaging • Pharmaceutical • Biomedicine	(*27*)
Bio-based polyethylene terephthalate (BioPET)	Made from ethylene oxide, a by-product of the oxidation of first-generation ethanol, which is obtained from starch and plant sugars	• Durable bottles • Biomedicine • Packaging	(*28*)
Bio-polyethylene (BioPE)	Made from first-generation ethanol by catalytic dehydration of the carboxylic acid group to ethylene	• Textile production • Toy production • Cosmetics • Food packaging	(*29*)

Polyhydroxyalkanoates (PHAs) are a type of polyester and one of the only bioplastics made entirely from a wide range of microbes in an unbalanced growth environment. This allows the microbes to store more intracellular carbon and energy reserve material (*30*). Based on the total number of carbon atoms in the side chains of the PHA monomer units, PHA has been generically divided into three forms: short-chain-length (SCL), medium-chain-length (MCL) and long-chain-length (LCL) PHA (*31*). However, research on MCL-PHA is limited since commercial PHA production requires expensive fermentation procedures, and no viable microorganisms produce MCL-PHA (*32*). A wide range of commercial uses of PHA can be attributed to its exceptional physicochemical properties. These include bioplastic films for crop protection, biodegradable disposable bottles, bioimplant materials, bone marrow scaffolds, orthopaedic pins, sutures, adhesion barriers, stents, repair patches, swabs, drug delivery carriers, biodegradable food packaging bags, automotive, infrastructure, aviation, space and military applications (*33*). Polyhydroxybutyrate (PHB) is the first microbial bioplastic discovered in the bacterium *Bacillus megaterium* (*34*). Nevertheless, its processing limitations, brittleness, hydrophilicity and insufficient mechanical and structural qualities limit its medical applications (*35*, *36*).

It is known that PHAs have been synthesised by more than 300 microorganisms, including bacteria such as *Wautersia eutropha, Cupriavidus necator, Thermus thermophilus, Hydrogenophaga pseudoflava, Saccharophagus degradans, Azohydromonas lata, Rhodobacter sphaeroides* and *Zobellella denitrificans* (*37*) and algae such as *Nostoc muscorum, Spirulina platensis, Synechococcus elongatus, Aulosira fertilissima, Botryococcus braunii* and *Dunaliella salina* (*38*). PHA is produced in five main steps: pretreatment, fermentation, harvesting, extraction from microorganisms and purification.

Sugars such as glucose or sucrose or other sugar-based substances such as maize, which contribute to high cost, have been largely used for PHA production (*39*). Thus, recent research has focused on the use of low-cost carbon substrates such as waste or wastewater to concurrently minimise PHA production and disposable waste costs (*40*). In this concept, many low-cost carbon sources such as lignocellulosic raw materials (wood, xylose, hemicellulose hydrolysates, wheat bran, *etc.*) (*41*), whey (hydrolysed soy and malt, hydrolysed whey and whey molasses) (*42*), molasses (sugar beet, cane sugar and soy molasses) (*43*), waste cooking oils (olive oil, coconut oil, soybean oil, palm oil, *etc.*) (*44*) and wastewater (brewery, palm oil, paper and food) were studied (*45*).

## PRODUCTION OF BIOPLASTICS FROM BIO-REFINERY WASTES

The numerous forms of waste generated by different industries, such as agricultural, industrial and municipal waste, are used to synthesise green plastic. Using organic waste as a feedstock in this process has dual benefits: it aids in waste management and helps reduce pollution from traditional plastics. Biopolymers are most typically produced by a method that incorporates microorganisms. Recently, algal biomass has been one of the most popular and environmentally friendly sources for the production of bioplastics and integration of advanced technologies like the Internet of Things (IoT) to improve production.

There are several challenges to utilising biorefinery waste for the production of bioplastics, even though it can produce biopolymers. The availability and amount of waste material is the most important factor. Secondly, it is important that the generated waste be biodegradable and have a constant composition. An extremely low moisture content is a prerequisite for transporting waste material to the production site. The commercial production of bioplastic (green plastic) from a range of biorefinery wastes requires an adequate and regular supply of the waste material (*46*).

### Agricultural waste

One advantage of using organic waste as a feedstock in this method is that it helps to reduce contamination from conventional plastics. Another advantage is that it contributes to waste control. Measures need to be taken to limit the increasing volume of agricultural waste. Every year, over 50 tonnes of agricultural waste are generated worldwide (*47*), which takes up about 28 % of the world’s agricultural land, equivalent to 1.4 billion acres of useable arable area (*48*, *49*). According to Heredia-Guerrero *et al*. (*50*), non-edible agricultural waste, which is created during agricultural processing, amounts to around 25 tonnes, according to the FAO data from 2013. China produced more grain than any other country, about 4.52 billion tonnes of manure from poultry and cattle, and about 3.03 billion tonnes of forest litter (*51*). According to Madurwar *et al*. (*52*), India produces about 3.5 million tonnes of agricultural waste per year from different sources. In 2016, approx. 2.07·10^7^ tonnes of waste were generated by the agricultural sector in the European Union (EU), which is 0.82 % of total waste produced in the EU countries. The pollution of soil and water sources can be caused by the simple disposal of this agricultural waste, which has a significant impact on the ecosystem (*53*).

#### Biorefinery based on lignocellulosic biomass

Plants are the main source of lignocellulosic materials such as cellulose, hemicellulose and lignin, together with trace amounts of starch, extractives, ash and proteins. Plant age, biomass, tissue and local environmental variables affect the concentrations of these chemicals. Lignin, which is found in plant biomass, is an important polymer that covalently binds cellulose and hemicellulose and consists of phenyl-propane units linked by non-hydrolysable bonds (*54*, *55*). The ether linkages (β-O-4) and chemical functional groups of lignin make it resistant to hydrolysis or microbial hydrolysis (*56*, *57*). However, its abundance in plant biomass makes it a sustainable and promising carbon source and a viable feedstock. Lignin production, predicted to be between 5 and 36·10^8^ tonnes per year, is a suitable carbon source for the ecologically friendly and cost-effective production of biopolymers.

The conversion of lignin to biopolymers involves two basic steps. First, the lignin polymer is broken down into constituent parts and depolymerised to yield a low molecular mass of lignin. The second stage is the production of bio-based products and opening lignin rings in microorganisms through the β-ketoadipate pathway. The molecular mass of lignin should be low enough to allow microbial bioconversion, and aromatic rings with a number lower than five should be able to pass through the cell wall and be digested by microorganisms (*58*).

The initial step in the bioconversion of lignocellulosic waste into bioplastics is to hydrolyse the lignocellulosic components from waste into fermentable sugars. The second step is detoxification of the hydrolysate and the removal of any inhibitory compounds generated during hydrolysis (*59*). Bacterial species such as *Pseudomonas* can form biopolymers by joining aromatic and aliphatic monomers in a series and valorizing various chemical compounds (*60*). Furthermore, different investigations have explored the possibilities of using *Rastlonia* and *Bacillus* sp. to bioconvert lignin into microplastics (*61*). Several studies have used *P. putida* KT440 to produce PHAs from lignocellulosic waste. The main advantage of using lignocellulosic waste over other substrates is their low carbohydrate content. One disadvantage of using lignocellulosic material as a carbon source is that it reduces microbial cell growth.

Lignocellulose, which contains lignin (20–30 %), cellulose (40–50 %) and hemicelluloses (20–50 %), is considered the most abundant and viable carbon source. Hemicellulose and cellulose are important sources of edible sugars, but lignin is not degradable due to its rich aromatic structure (*62*). Cellulose consists of β-1,4-glycosidic linkages of d-glucose and is neither digestible by humans nor soluble in water due to its large molecular mass and crystalline structure. Hemicellulose is an amorphous polysaccharide consisting of several pentose sugars, including xylose, galactose, rhamnose, mannose and arabinose. As a result, cellulose and hemicelluloses are separated from lignin by a process known as delignification, which provides a high sugar yield for microbes to produce PHA. The basic method for degrading solid biowaste into simpler sugar monomers is to pretreat it with cellulase enzymes and then steam it with diluted acids (*63*). *Pseudomonas cepacia* synthesised 48–56 % of poly-3-hydroxybutyrate with a yield of 0.11 g per g of xylose. However, the engineered *E. coli* carrying *phbC* genes of *Ralstonia eutropha* was able to synthesise 74 % PHB with a yield of 0.226 g per g of xylose and was thus superior to the indigenous strain.

However, genetically modified *E. coli* carrying *Ralstonia eutropha phb* can produce 74 % PHB with a yield of 0.226 g per of xylose, outperforming the indigenous strain (*64*, *65*). It has also been investigated whether hemicellulose hydrolysates can be used directly as a sugar combination. Furthermore, the results showed that *R. eutropha* can ferment small amounts of hemicellulose hydrolysates, primarily for cell development, using fed-batch fermentation. Recently, the growth conditions of *R. eutropha* 5119 have been improved to produce PHA using lignocellulosic biomass hydrolysates (LBHs), such as *Miscanthus* biomass hydrolysate (MBH), barley biomass hydrolysate (BBH) and pine biomass hydrolysate (PBH), as effective carbon substrates. In addition, indigenous yeast species like *Wickerhamomyces anomalus* VIT-NN01, *Candida tropicalis* BPU1 (*66*) and marine *Pichia kudriavzevii* VIT-NN02 (*67*) have been reported to produce PHA using sugarcane molasses with palm oil, banana peel hydrolysate and orange peel, respectively, as the main carbon substrate.

#### Spent coffee grounds

Considering that coffee is one of the most popular consumer commodities in the world market today, it is not surprising that the coffee industry generates a large amount of waste through the production process. Furthermore, the coffee manufacturing sector is one of the fastest growing in the current food market. Around 6 million tonnes of waste coffee grounds are produced annually (*67*).

The age of the coffee plants, their location, the environment in which they grow, and the quality of the soil affect the chemical composition of spent coffee grounds. Coffee contains nitrogenous compounds (amino acids, a building block of proteins), lipids (free fatty acids, triglycerides and sterols), crude fibre (cellulose, hemicellulose, lignin, mono, oligo- and polysaccharides) and a variety of minerals. The traces of bioactive compounds in spent coffee grounds, such as polyphenols, diterpenes and alkaloids (trigonelline, caffeine), contribute to their antibacterial, anticarcinogenic and antioxidant properties (*68*-*72*).

Cellulose (7–9 %), hemicellulose (32–42 g/L), lignin (0–26 g/L protein (10–18 g/L), lipids (2–24 g/L), caffeine (0–2 g/L) and ash (1–2 %) are the main constituents of spent coffee grounds. Several pretreatment methods can dissolve the carbohydrates in spent coffee grounds. The primary by-products of the hydrolysis of spent coffee grounds are galactose, mannose and arabinose (*73*-*75*). *Bacillus subtilis*, *Bacillus firmus*, *Pseudomonas fluorescens*, *Burkholderia cepacia, Burkholderia sacchari, Novosphingobium nitrogenfigens* Y88 and *Halomonas halophila* are among the microorganisms that can break down the monosaccharides in spent coffee grounds to produce PHA (*76*-*82*). In a study by Kovalcik *et al*. (*82*), spent coffee ground hydrolysate, which is rich in carbohydrates (mannose, galactose and arabinose), was used to produce PHA with *Halomonas halophila* CCM 3662. In their study, dried spent coffee grounds were defatted, phenolic compounds were adsorbed onto Amberlite XAD4 and finally acid/alkali-treated to obtain spent coffee ground hydrolysate rich in sugars, which was later used to obtain PHB titres of 0.95 g/L with a yield on dry cell mass basis of 27 %.

**Fig. 1:**
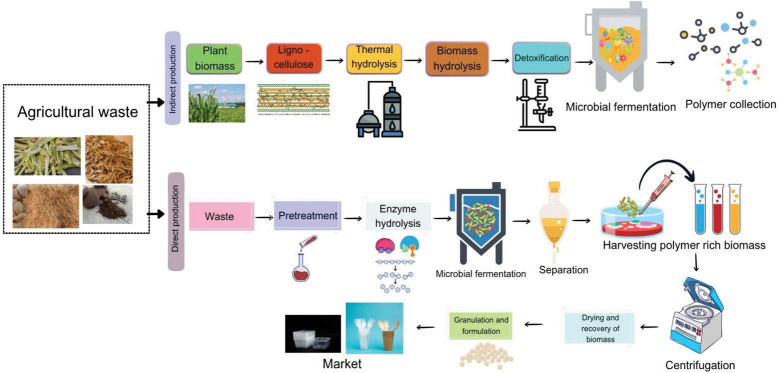
Production of bioplastics from agricultural waste (Canvas was used as the primary design tool)

According to a study in which *C. necator* was compared with different oils and the oil from spent coffee grounds, the oil extracted from spent coffee grounds produced significantly more effective PHB (*83*). The dry cell mass increased to 55 g/L and the PHB content to 89.10 % when the oil from spent coffee grounds was used in fed-batch mode. Because the oil from spent coffee grounds had a higher free fatty acid content than the other oils tested, it formed significantly more PHB. The main disadvantage of the oil extracted from spent coffee grounds is that it foams, although this can be reduced by using other oils that act as both carbon sources and anti-foaming agents. In another study, a recombinant bacterial strain called *Cupriavidus necator* DSM 428 was used, which utilised the oil from spent coffee grounds as a substrate and a fed-batch fermentation process to produce carbon dioxide. The final product had a PHB concentration on dry biomass basis of 10.7 g/L. An overview of a study on the agricultural waste is shown in Fig. 1.

### Industrial waste

The public is becoming increasingly aware of plastic waste derived from petroleum. PHAs and other bio-based and easily biodegradable polymers are reducing the market share of petroleum-based plastics that are difficult to break down (*84*). Many different types of bacteria produce naturally occurring PHAs in response to unbalanced growth conditions, such as nitrogen limitation (*85*). PHB is the most well-known PHA, widely used in various industries, including packaging, vehicle components and household appliances. It is a bio-based thermoplastic polyester with many favourable properties of polypropylene (*84*). Unlike other bioplastics, such as polylactic acid, PHB has the potential to rapidly biodegrade to carbon dioxide and water (*86*).

Alternatively, bioplastics could be made cheaper and more environmentally friendly by reusing or recycling waste products from agro-processing industries, such as sugarcane molasses and whey from the food industry, which are abundant and have low to zero disposal costs.

#### Cheese whey

The global dairy industry faces the challenge of finding a cost-effective and environmentally responsible method of eliminating waste whey, a by-product of cheese and yoghurt. Large dairy producers can use sweet whey (pH=6–7) to produce whey protein and lactose powder. Nevertheless, the dairy industry faces significant challenges due to the presence of lactic acid in acid whey, which is produced in cottage cheese and Greek yoghurt. Dairy producers must pay for the removal of more than 4 million tonnes of acid whey produced in the United States, most of which is either disposed of in wastewater treatment plants or on fields (*87*). Each year, American dairy farms dispose of more than 4 million tonnes of acid whey; most of this waste ends up in wastewater treatment plants or on fields. As a result, acid whey causes logistical, financial and environmental problems for the dairy industry. Lactose (38–49 g/L), lactate (5.1–8 g/L), lipids (about 1.1 g/L), proteins (4.2–10 g/L) and mineral salts (2.6–5.1 g/L) are the typical components of acid whey. The production of PHAs from whey lactose and lactate would benefit the environment and offer a cheap, renewable feedstock that would not compete with edible goods. Although acid whey can potentially be used as a feedstock for bioplastics, several processing issues make it challenging to develop efficient techniques. For instance, two of the main carbon sources, lactose and lactic acid, are inaccessible to natural microbes. Lactose must be pretreated in acid whey to produce sugars with a higher carbon-to-nitrogen ratio (C/N) and promote fermentation, which is problematic. PHB production increases with higher C/N ratio and some industrially relevant bacteria cannot ferment lactose. The nitrogen retention of whey and acid whey reduces the C/N ratio. A higher C/N ratio (14:1) tends to promote biomass growth, but at the same time, the cells do not store as much PHB as they would with a lower C/N ratio (*e.g.* 2:1, resulting in a higher PHB content but a lower total yield). Therefore, for a higher PHB yield, a C/N ratio of 14:1 is better. If higher PHB content in the cells is needed, a lower C/N ratio (2:1 with bovine serum albumin (BSA)) is better.

**Fig. 2:**
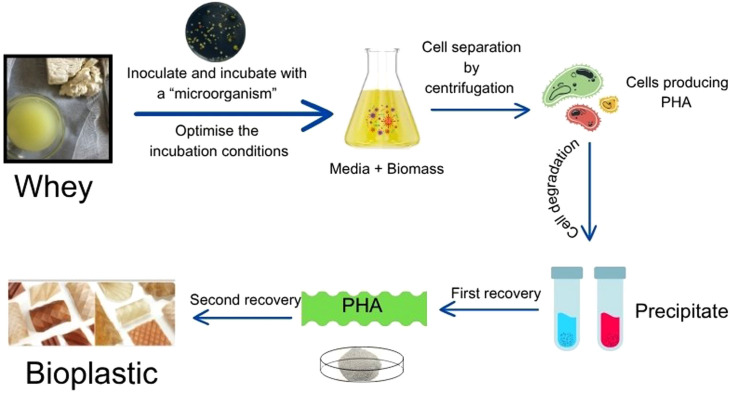
Production of polyhydroxyalkanoates (PHA) from acid whey (Canvas was used as the primary design tool)

For some microorganisms to successfully convert whey to PHAs, lactose must be broken down into monosaccharides by hydrolysis. The second main impediment to the use of acid whey is the low pH created by lactic acid, which inhibits most microorganisms that produce PHA. The inhibition induced by lactic acid reduces productivity, economic viability and the ability to fully utilise carbon in the production of PHA from acid whey (Fig. 2). In addition, keeping the fermentation pH˃7 and the PHA synthase enzymes active is difficult due to the formation of acidic intermediates and the low initial pH of acid whey.

The first step in evaluating the effects of different conditions on PHB synthesis in *E. coli* LSBJ with plasmid pBBRSTQKAB using synthetic acid whey was to determine the carbon and nitrogen supply, the initial pH and the addition of minerals and salt. This made it possible to determine if and when a decrease in the pH blocked the PHB biosynthetic pathway and what effect a stable pH has on PHB production. Finally, to maximise PHB production, the amounts of carbon sources, initial pH, minerals and salts in raw acid whey must be adjusted (*88*). Although recombinant *E. coli* LSBJ produced high PHB yields on various substrates and some *E. coli* strains are acid tolerant, it was hypothesised that this would be a viable method for converting acid whey to PHB. A high C/N ratio can, in principle, lead to changes in metabolic flow and increase the concentration of PHB. The production of PHB could be increased by the addition of salts and minerals. In summary, the results will show that the creation of a sustainable system that utilises waste with negative costs to produce biodegradable plastic precursors is possible.

Another cost-effective biotechnological method for PHA synthesis is the use of mixed microbial consortia (MMC), which eliminates the sterilisation phase. MMC is described as a microbial community of unknown composition that is capable of carrying out certain intra- and extracellular processes. As a result, the activated sludge produced in wastewater treatment plants is classified as MMC. The use of mixed microbial cultures is usually carried out in a series of stages, depending on the substrate type.

The first phase is acidogenic (anaerobic) fermentation, which produces volatile fatty acids (lactic, acetic and propionic acid) from carbon-rich wastewater. Following the production of volatile fatty acids, the culture was enriched mostly under aerobic dynamic feeding conditions in a sequential batch reactor to yield biomass with PHA accumulation potential (*89*). Another stage is PHA formation (accumulation) in biomass to maximise PHA content (*90*). The PHA was then removed and purified in the final stage (*91*). This production procedure can be used for complicated substrates such as olive mill effluent, cheese whey and other food waste to produce more homogeneous PHA (*92*).

#### Sugar cane

Polyhydroxyalkanoates, known as PHAs, are an alternative to petroleum-based polymers. Microbes that thrive in an unbalanced nutrient environment turn PHAs into biodegradable and biocompatible bioplastics for energy storage. PHAs are divided into numerous types based on their chemical composition. Polyhydroxybutyrate (PHB) and 3-hydroxybutyrate (3HB) are two homopolymers consisting of a single monomer. Polypropylene (PP) and polystyrene (PS) are petroleum polymers, and PHB plastics have comparable structures; they are both hard and brittle. Nevertheless, the production cost of PHAs is still higher than for polymers derived from petroleum. The production method has some pitfalls that can increase PHA costs.

To reduce the cost of producing PHA, one of several readily available and inexpensive raw materials can be used. Sugarcane molasses is a highly exploited carbon source. A by-product of sugar refining, sugarcane molasses contains a high concentration of sugars such as sucrose, fructose and glucose. It also contains trace amounts of essential elements for cell formation, including organic acids, amino acids, vitamins and minerals.

*Halomonas* sp. are bacteria that produce PHB. The rod-shaped bacterium *Halomonas* is Gram-negative. This strain is halotolerant, which means it can thrive in environments with NaCl mass per volume ratios ranging from 0.1 to 32.5 %. To avoid cross-contamination, media with high NaCl amounts should be used when growing *Halomonas*. *Halomonas* is therefore a suitable strain for PHB production. Based on its dry cell mass, this strain can accumulate 7-80 % PHB. Furthermore, unlike other strains, it can metabolise sucrose independently, which means it does not require hydrolysis to utilise it. These advantages make *Halomonas* sp. a good bacterium for PHA production. It may be possible to determine if *Halomonas* sp. can produce PHB from cheap substrates such as sugarcane molasses.

The most successful strain of *Halomonas* for metabolising sugarcane molasses and producing PHB was found to be *Halomona scerina* YK44. The cultivation conditions were adapted to increase PHB production. The NaCl mass per volume ratio was 2 % and the pH was 7. With 8.65 g/L dry cell mass, 6.55 g/L PHB and 76.55 % PHB, the strain YK44 was the best producer of PHA at 4 %. The addition of a cost-effective nitrogen source improved cell proliferation and PHB accumulation; this is paramount for microbes because the C/N ratio influences PHB production (*93*). Additionally, the inhibitory effect of sugarcane molasses was discovered. By comparing the PHB film produced from sugarcane molasses to a film produced from simple sugars and petroleum-derived polymers such as polyethelene terephthalate (PET), polybutylene terephthalate (PBT), PP and PS were able to confirm its application and investigate its physical and thermal properties. This made it possible to evaluate the potential for substitution.

### Municipal waste

The increasing amount of wastewater generated as a result of both population and economic growth has exacerbated the already acute water shortage in the world. Jones *et al*. (*94*) estimated that global wastewater production is 359.4·10^9^ m^3^ per year. Water reuse has proven to be one of the most promising techniques for solving this problem (*95*-*97*). Graywater, which is used water from various taps, showers, baths and kitchen sewage, has the potential to be reused (*98*).

**Fig. 3:**
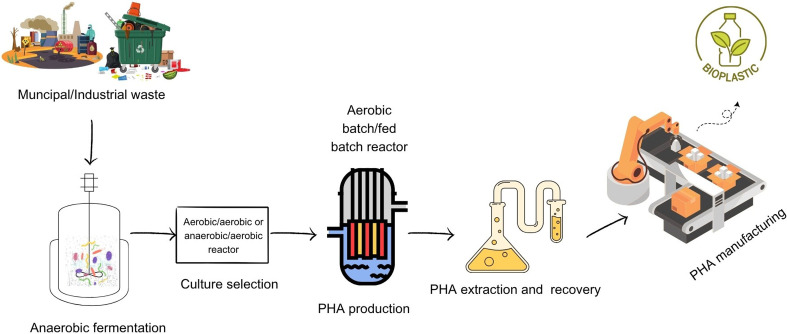
Process of production of bioplastics from municipal or industrial waste (Canvas was used as the primary design tool)

To safely discharge or reuse blackwater, it must be thoroughly treated to remove toxins and other harmful contaminants. Water that does not come from a toilet is treated separately, as opposed to graywater, which includes wastewater from sinks, showers and laundry. There is a significant risk of bacterial, viral and pathogenic contamination when blackwater is used in chemical production. Any water or effluent that could have an impact on human health, including sewage, must first be treated before being used in industries that produce food, grow crops or provide potable water (*99*). PHAs produced by various bacteria and archaea from different carbon sources have recently attracted interest due to their potential environmental friendliness (*100*). Bioplastics derived from raw sewage and household peels are a revolutionary upcycling approach that can considerably reduce the cost of wastewater treatment (Fig. 3).

#### Sewage

According to the International Water Association's 2018 wastewater report, industrialised nations can dispose of 70 % of their sewage, while poor countries only manage 8 % (*101*). The growing amount of sewage sludge produced by various effluent treatment plants is a cause of great concern, as it is difficult to dispose of it responsibly.

Algal growth media have improved the models for algal production, because they can grow in urban environments or on non-land structures such as columns, and purify this water. Microalgae cultivated in nutrient-free wastewater have a high protein content, making them a viable candidate for conversion into a low-impact bioplastic (*102*).

*Arthrospira platensis* and *Chlorella* sp. are two examples of microalgae with a high protein content, which ranges, on dry mass basis, from 46 to 63 %. Blends with petroleum plastics or bioplastics are the primary focus of research into the use of microalgal biomass in the development of bioplastics (*103*). The development of microalgae in wastewater has several advantages over standard plant and seed cultivation methods, including the elimination of soil and the use of non-potable water as a growth medium. Their CO_2_ fixation rates are faster than those of other, more complex species, and they can reportedly triple their biomass in just a few hours (*104*). Because of their small size and high protein content, they can be converted directly into polymers without previous treatment, increasing the cost-effectiveness of large-scale production while reducing waste (*105*).

#### Domestic peel

Fruit and vegetable waste has a high potential for the production of microbial bioplastics such as PHA. In one study, for example, *C. necator* and *P. citronellolis* were used in co-culture to produce poly(3-hydroxybutyrate [P(3HB)] and PHA, with apple pulp waste serving as the sole carbon source for the bacteria (*106*). The researchers discovered that the combination of P(3HB) and PHA lead to comparable quantitative results to develop flexible and elastic films. *Pseudomonas citronellolis* produced 1.2 g/L of PHA when grown on the soluble fraction of apple pulp.

Another study used *Bacillus* sp. to produce PHA from hydrolysed apple pomace fatty acids as a carbon source (*107*). The production of biodegradable bioplastic films can be facilitated by using banana peel, which is high in starch and readily available in large quantities. The most important parts that are discarded are banana peels and cellulosic fibres. That study provides a new viewpoint on converting waste biomass into lucrative products. The new component of that study was the use of waste biomass to produce a bioplastic film suitable for the packaging of dry goods. The use of cellulose fibre as a filler improved the physical, mechanical and thermal properties of the bioplastic film. Although the peel and the pseudostems are wasted, they could be recycled and used to make the packaging for dry goods. To do this, the following subactivities were completed (*108*): firstly, the availability of starch, a complex carbohydrate, can be determined by analysing the proximate composition of the banana peel. The starch can then be extracted and characterised. The optimal parameters can be determined to maximise yield. Fibre from banana pseudostem is extracted and characterised. Water absorption rate, tensile strength and elongation at break of the produced bioplastic film are improved. The physicochemical parameters of the synthesised plastic film are determined, including solubility, transparency, thickness and density. The best film is then analysed using Fourier transform infrared spectroscopy (FTIR), differential scanning calorimetry (DSC) and thermogravimetric analysis (TGA), which allowed a clear comparison of the bioplastic film with and without cellulosic fibre.

## ALGAL BIOMASS

The accumulation of huge amounts of plastic waste generated by the increasing use of plastic materials in the modern world has harmed ecosystems and human health, especially during the COVID-19 outbreak (*109*, *110*). Most modern plastics are made from petroleum-based polymers and do not degrade in the environment. As a result, plastic waste ends up in landfills for hundreds of years after it has dissolved (*111*). Thus, the environmental benefits of bioplastics, renewable and biodegradable alternatives to petroleum-based plastics, have recently received much attention (*112*). Numerous methods and materials are available to produce bio-based plastics, each with advantages and disadvantages in the development of ecologically suitable replacements. Bioplastics comprise polysaccharides, proteins and lipids. These three components give bioplastics their distinct properties, which makes them valuable for various applications.

Bioplastics are a revolutionary step towards more sustainable polymers, as they are made from renewable raw materials. Bioplastics are now produced from various biomaterials, including corn, potatoes, sugarcane, banana peels, agricultural waste, algae, vegetable oils, wood, food scraps and various cereal crops (*113*). The wide range of sources used for bioplastics indicates their versatility, which is consistent with the greater goal of sustainable material sourcing.

Polylactic acid (PLA), poly-3-hydroxybutyrate (PHB), organic polyethylene (PE) and starch-based variants are the current market leaders among bioplastics (*114*). Because of their different properties, each type is well suited for different applications in numerous fields. However, the development of bioplastics from seaweeds (macroalgae) is a fascinating discovery that is still a work in progress. This latest invention, which demonstrates the ongoing development of sustainable materials, has the potential to significantly improve the environmental effect of the plastics industry. Bio-based plastics offer a more sustainable future for materials that are essential to our daily lives, and they are continuously developed in response to continuous research and innovation.

Algae are various living organisms, including unicellular and multicellular photosynthetic organisms. Bioplastics are made from several algal by-products. *Spirulina* biomass is extensively used for the production of bioplastics, although it is difficult to harvest. However, macroalgae, such as seaweeds, offer certain advantages over microalgae. Seaweeds produce a large amount of biomass, are inexpensive, can grow under various conditions and are easy to manage and collect in their natural habitat. Seaweeds produce polysaccharides that are widely used in microbiology, food technology, biotechnology, medicine and, more recently, in the plastic industry. Bioplastics are made from seaweed polysaccharides, which are inexpensive, environmentally friendly and non-toxic. Bioplastics obtained from seaweeds have a high tensile strength and are now far superior to conventional plastics (*114*, *115*).

### Macroalgae

Seaweeds, also known as macroalgae, can vary in shape and size depending on water depth. The enormous diversity of algae and the classification according to their pigmentation, especially to red (the taxa Rhodophyta), brown (the taxa Phaeophyta) and green algae (the taxa Chlorophyta), provides a comprehensive basis for understanding them. Brown macroalgae, known as Phaeophyta, are found in shallow coastal waters worldwide. Fucoxanthin pigments (*116*) give them their unique brown colour. Because of their high polysaccharide content, they are good raw materials for bioplastics (*117*). Phaeophyta species produce and store a wide range of secondary metabolites that have the potential to be used as bioplastics.

#### Process for the production of bioplastics from macroalgae

Macroalgae are collected by various approaches, including mechanical harvesting and handpicking (*118*, *119*). Collecting macroalgae from the beach by hand is very tedious. This strategy is effective for localised work but impractical for mass production (*119*). In contrast, mechanical harvesting involves gathering macroalgae from water using boats equipped with specialised equipment.

The extraction of polysaccharides from macroalgae is an important step in the production of bioplastics from these plants. Extracts from brown macroalgae include alginate and laminarin, while carrageenan is obtained from red macroalgae and ulvan from green macroalgae (*120*-*122*).

Alginate films are usually produced by casting and solvent evaporation (*123*). Unlike films made with alginate polymers ionically crosslinked with Ca^2+^ ions, sodium alginate films have lower mechanical properties, barrier properties and water resistance (*124*, *125*). The most common ionic crosslinking processes are external, internal, interfacial and direct mixing of the crosslinking agent (*126*). Covalent crosslinking with substances like citric acid and ferulic acid has improved thermal stability, elasticity and transparency of alginate films (*127*).

Macroalgal bioplastics are produced using methods such as extrusion blow moulding, thermoreversible gelling, casting and compression moulding. Plasticizers such as glycerol are widely used to increase malleability (*128*). The polysaccharide powder is dissolved by heating and stirring the liquid. The powder is dissolved in water, then poured into a mould and left to cool and harden. The physical properties of hydrogel films can be modified by ionic or covalent crosslinking of polymers such as alginate and carrageenan with multivalent cations such as calcium. By blending different algal polysaccharides, the properties of bioplastics can be modified for various applications, from food packaging to medical equipment (*129*, *130*).

### Microalgae

Microalgae have emerged as a promising feedstock for bioplastic production due to their rapid growth rates, high photosynthetic efficiency and the ability to sequester atmospheric CO_2_. Additionally, their biomass contains substantial amounts of lipids, carbohydrates, proteins, cellulose, hemicellulose and lignin, which serve as precursors for various types of bioplastics. As a result, microalgae are well-suited for producing high-quality bioplastics, including those based on starch, cellulose, polyhydroxyalkanoates (PHAs), polyhydroxybutyrate (PHB), polylactic acid (PLA), polyethylene, polyvinyl chloride (PVC) and protein-derived polymers.

Although several natural resources are available for the production of bioplastics, a large proportion of these resources will exacerbate the already difficult food situation. The production of bioplastics from these components can harm the agricultural sector. For this reason, microalgae have become a popular source for the production of bioplastics. This type of microalgae is good for the production of bioplastics because it is easy to cultivate, requires little feed and a favourable growth environment, and grows quickly.

Compared to other materials, bioplastics offer higher mechanical and tensile strength. Microalgae produce biopolymers such as PHA and PHB intracellularly that can be used in bioplastic production (*130*). Microalgae have excellent properties, such as quick growth and easy synthesis, which makes them a potential source for bioplastics.

Certain *Spirulina* species can use photoautotrophic respiration to produce biopolymers like PHA and PHB. It can also be used as a filler or a reinforcing fibre in bioplastic composites. By incorporating microalgae into biocomposites, the mechanical properties of bioplastics can be improved (*131*).

*Chlorella* bioplastic often has a low melting point. The melting point of the plastic can be increased by adding a compatibilizer. An ultrasonic homogenizer is used as a pretreatment to increase the mechanical properties and tensile strength of the *Chlorella* PVA bioplastic. The addition of *Chlorella* as a filler to PVC improves its mechanical properties (*131*).

#### Production process of bioplastics with microalgae

Bioplastics derived from microalgae are the result of a fascinating and complex technology that uses the power of these bacteria to make environmentally friendly products. Selecting microalgae, cultivating them, harvesting them, extracting their lipids, synthesizing bioplastics, characterising them, and finally, utilising their waste are all common processes in the production of bioplastics from microalgae. The method starts with the careful selection of microalgal strains known for their high biomass and lipid concentration. These strains are the main source of bioplastic precursors. Controlled habitats, such as ponds or specialised bioreactors, provide optimal conditions for the development of microalgae. The light intensity, temperature and nutrient contents are all closely controlled to ensure the maximum potential.

When the microalgal biomass reaches a certain level, they are harvested. The microalgae are removed from the growth medium by various methods, including centrifugation, filtration and flocculation. Lipid extraction is the process of obtaining usable lipids from microalgae. These lipids serve as raw ingredients in the production of bioplastics. The lipids are converted into bioplastics using various polymerization processes. Polyhydroxyalkanoates (PHAs) are a common type of biodegradable polymer produced from these lipids.

*Haloferax mediterranei* utilises carbohydrates as a carbon source to produce PHAs. Simulants of hydrolysates from seven different macroalgal biomasses were produced and PHA synthesis was investigated. The medium containing green macroalgae had the highest biomass concentration and PHA content. Growing *Haloferax mediterranei* in 25 % *Ulva* sp. hydrolysate at 42 °C and an initial pH=7.2 resulted in the highest cell dry mass and PHA concentration of (3.8±0.2) and (2.2±0.1) g/L, respectively. Poly(3-hydroxybutyrate-co-3-hydroxyvalerate) was the primary PHA constituent.

## ENGINEERED MICROORGANISMS AND PHAOME

**Fig. 4:**
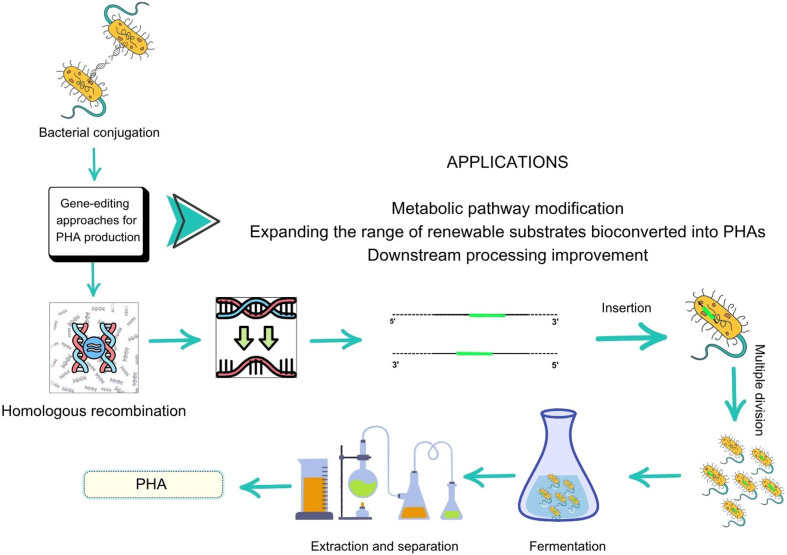
Genetically engineered strategy for PHA production (Canvas was used as the primary design tool)

Due to complex manufacturing processes, large-scale production and commercial applications of bioplastics may be hindered (*132*). PHA production from wild-type bacteria requires a complex extraction technique to recover PHA (*133*). Furthermore, synthesis from wild-type bacteria does not lead to a high yield. As a result, recent research has focused on the production of PHA using genetically modified bacteria, which can enable rapid growth, high cell density, simplified separation and lower costs for bioproduction (*134*). Bacteria can produce PHAs with a wide range of molecular mass, monomer configurations and ratios (*135*). Genetically engineered bacteria can produce single selected monomers instead of a mixture of copolymers (*136*). It is also called the ’PHAomeconcept’. This method produces specific PHA structures and constant molecular mass of bacteria (*135*). Recent improvements in designed microbes have improved PHA biosynthesis through ribosome-binding site (RBS) optimisation, promoter engineering, chromosomal integration, cell morphology engineering and reprogramming of cell growth behaviour (*137*). Considering PHA production from ’waste’ and ’engineered microorganisms’ together, the most innovative strategy proposed to merge these two production processes in a techno-economic way (Fig. 4).

## ROLE OF INTERNET OF THINGS AND MACHINE LEARNING

A promising area for the development of algae-based bioplastics is the integration of the internet of things (IoT) and artificial intelligence (AI), which can significantly improve the sustainability and productivity of microalgae and seaweed cultivation efforts (*138*-*141*). A combination of the IoT and machine learning (ML) enables accurate control and real-time monitoring of growing conditions, as well as predictive modelling and data-driven decision making. These technologies drive the shift towards smart and sustainable farming practices and are in line with the larger objectives of circular economy.

Both Khor *et al*. (*142*) and Ariawan and Makalew (*143*) emphasise the potential of the IoT to improve microalgal agriculture. By controlling and monitoring growth parameters like pH, temperature and light intensity in real time, Khor *et al.* (*142*) show how to achieve maximum biomass density on day 8 of cultivation. The IoT also enables remote monitoring and control of these parameters, which helps to increase the efficiency of the production of algae while reducing the need for human intervention. Ariawan and Makalew (*143*) developed a smart microfarm that monitors key environmental parameters for *Spirulina* growth, including water temperature and UV intensity. The importance of real-time monitoring and optimisation of microalgal growth conditions is evident in both studies by the continuous collection and management of data through cloud services.

The use of ML, a branch of AI, has been successful in improving microalgae farming methods. The study conducted by Lim *et al.* (*138*) proves that microalgae can be correctly identified and categorised with an accuracy of over 90 %. The production of bioplastics can be greatly improved by effectively identifying and utilising algal strains with suitable properties. Using ML to predict desirable traits and select strains with the best bioplastic production capacities can optimise strain selection (*144*, *145*). This leads to more efficient strain selection and contributes to production optimisation. In addition, ML can help predict microalgal growth, enabling better production forecasting and resource optimisation. To maximise production while minimising waste and costs associated with resource over- or undersupply, predictive models can be used to maximise resource optimisation (*146*).

## CIRCULAR BIOECONOMY

The transition to a circular bioeconomy and replacing traditional plastics have sparked the interest of manufacturers, politicians, and decision-makers worldwide in goods and materials made from bioplastic (*147*). Global industrial capacity will be expected to reach approx. 2.62 million annually. Sustainable development goals set by the European Union for 2020 predict a 4.7 million tonne increase in the global capacity to produce bioplastics by 2027, bringing the total to about 6.3 million tonnes. The fundamental challenge for EU members is the difficulty of scaling up advanced biorefineries that use known technologies to produce and sell high-quality bioproducts. The plastic supply chain must use circular economy principles and technology to reduce plastic waste and its environmental impact. Improving energy efficiency in the production of plastics and bioplastics while using renewable energy, developing products that can be reused and recycled, significantly reducing plastic consumption, increasing collection rates and penetrating markets with robust and circular recycling and 'upcycling' methods are all positive steps towards a future circular economy.

**Fig. 5:**
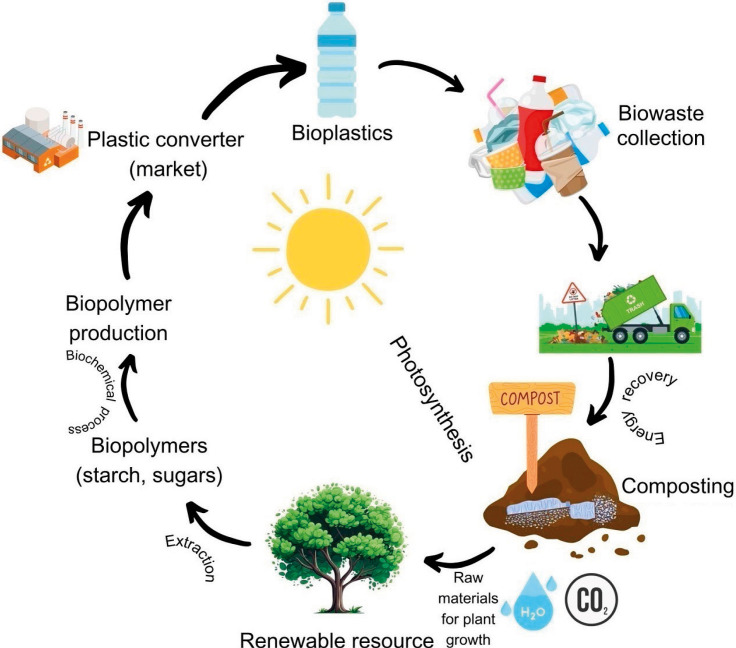
Life cycle of bioplastic towards a circular economy (Canvas was utilized as the primary design tool)

The 'circular plastic economy' (represented by green arrows) produces and recycles plastic waste using renewable energy and converts it into raw materials at the end of its life. All polymer products have a clearly defined circular end-of-life scenario (Fig. 5) and their starting materials are renewable (pyrolysis oils and lignocellulosic biomass). It can be defined by two guiding principles: better conversion to products increases the value of raw materials, and responsible product design reduces service time loss. By constantly maximising the value of goods, components and resources and minimising waste, circularity aims to improve the sustainable flow of biological and technological resources, such as agricultural waste.

## CONCLUSIONS

Plastic pollution in the oceans is a growing problem and researchers are under pressure to find solutions, such as efficient recycling processes for biodegradable plastics or viable alternatives to non-biodegradable conventional plastics, as these materials are increasingly discarded worldwide and fossil fuels are being depleted. There is a shift away from petrochemical plastics and towards bio-based polymers. The production of polymers from fossil fuels has a significant effect on the environment. Thus, bioplastics are predicted to continue to grow as a viable option. The main obstacle to the production and use of bioplastics is their cost-effectiveness. However, due to their benefits, the microbial production of bioplastics is becoming increasingly popular.

The main barriers to sustainable biopolymer synthesis are reducing the cost of the carbon source and improving production and extraction efficiency. In addition to lowering production costs, this research has shown that using different waste streams as a carbon source can close the loop of material consumption cycles, which is an important component of a circular bioeconomy. Global problems are addressed efficiently by manufacturing bioplastics as part of circular bioeconomy strategy. This technology is very promising for a sustainable economy and environment as it reduces the demand for finite resources.
